# Sweating the small stuff: A meta-analysis of skin conductance on the Iowa gambling task

**DOI:** 10.3758/s13415-019-00744-w

**Published:** 2019-09-06

**Authors:** Boban Simonovic, Edward Stupple, Maggie Gale, David Sheffield

**Affiliations:** Department of Psychology, University of Derby, Derby, UK

**Keywords:** Decision-making, Emotion, Reward

## Abstract

To systematically examine the role of anticipatory skin conductance responses (aSCRs) in predicting Iowa Gambling Task (IGT) performance. Secondly, to assess the quality of aSCR evidence for the Somatic Marker Hypothesis (SMH) during the IGT. Finally, to evaluate the reliability of current psychophysiological measurements on the IGT. Electronic databases, journals and reference lists were examined for inclusion. Data were extracted by two reviewers and validated by another reviewer, using a standardised extraction sheet along with a quality assessment. Two meta-analyses of aSCR measures were conducted to test the relationship between overall aSCR and IGT performance, and differences in aSCR between advantageous and disadvantageous decks. Twenty studies were included in this review. Quality assessment revealed that five studies did not measure anticipatory responses, and few stated they followed standard IGT and/or psychophysiological procedures. The first meta-analysis of 15 studies revealed a significant, small-to-medium relationship between aSCR and IGT performance (r= .22). The second meta-analysis of eight studies revealed a significant, small difference in aSCR between the advantageous and disadvantageous decks (r= .10); however, publication bias is likely to be an issue. Meta-analyses revealed aSCR evidence supporting the SMH. However, inconsistencies in the IGT and psychophysiological methods, along with publication bias, cast doubt on these effects. It is recommended that future tests of the SMH use a range of psychophysiological measures, a standardised IGT protocol, and discriminate between advantageous and disadvantageous decks.

## Introduction

Learning and decision making in uncertain or ambiguous situations is an important feature of everyday life. Even simple decisions have a potentially perplexing array of options that need to be evaluated to make an optimal choice. Blanchette and Richards (2010) argue that the decision-making process involves several stages: extracting meaning from ambiguous information in order to construct a mental representation (interpretation process); evaluating the evidence, estimating the value and likelihood of the occurrence of differing outcomes (judgement); and finally, drawing inferences and selecting from the available options (choice). Among the most important questions in this field is how emotion shapes different forms of decision making (Quartz, 2009). Often emotion was seen as a disruptive factor (Reimann & Bechara, 2010); however, since Damasio’s (1994) ground-breaking Somatic Marker Hypothesis (SMH), emotion has been viewed as playing a significant role in the judgement of value that drives decisions. The theory postulates that the foundation of optimal decision making rests on the positive or negative emotional reactions to previous outcomes of choices, rather than rational, cognitive calculation of gains and losses. It is assumed that these emotional reactions guide decision making by creating positive or negative somatic markers (Bechara, Damasio, & Damasio, 2000). According to Damasio (1994), in situations of ambiguity and uncertainty, these somatic markers create an emotional signal about the “goodness” or “badness” of choices. Only those options that are marked as good are then considered for selection. Thus, it is assumed that somatic markers pre-empt or guide cognitive, reason-based choice.

A strength of the SMH rests on the specification of its neural architecture. Damasio (1994) argued that somatic states can be generated from primary (the "Body Loop") and secondary (the "As-if Body Loop") inducers. Primary inducers are innate or learned stimuli that induce unpleasant or pleasurable states. They usually elicit an automatic response through the amygdala. For instance, seeing a snake would trigger a critical substrate in the neural system connected to the amygdala and induce an obligatory fight or flight response. Conversely, secondary inducers of somatic states are generated by thoughts and memories of a hypothetical state (i.e. a memory of seeing a snake). The recalled memory also induces automatic, involuntary responses, but contrary to the primary inducers, the responses are generated through the medial prefrontal cortex (MPFC), amygdala, insular cortex, somatosensory cortex, brainstem nuclei and the ventromedial prefrontal cortex (VMpfc) (Bechara, 2001). A number of brain imaging studies provided contrasting evidence in support of the SMH (e.g. Heims, Critchley, Dolan, Mathias, & Cipolotti, 2004; Lawrence, Jollant, O’Daly, Zelaya, & Phillips, 2008; Wilder, Weinberger, & Goldberg, 1998) providing evidence for the MPFC, dorso-lateral prefrontal cortex (DLPFC) and insula involvement during IGT performance; however, not from the proposed main “loops” suggested within the SMH (e.g. Bechara, 2001). Nevertheless, the aforementioned brain imagining studies did not include other brain regions associated with learning probabilities in uncertain conditions (e.g. Huettel, Song, & McCarthy, 2005; Lin, Chiu, Cheng, & Hsieh, 2008), thus focusing only on the VMpfc systems in relation to IGT performance.

The Iowa Gambling Task (IGT) is often used as an experimental tool for assessing the SMH (Bechara, Damasio, Damasio, & Anderson 1994). It is argued that the IGT resembles real-life decision making and is characterized by the uncertainty of punishment and reward schedules (Bechara et al., 2000). During the IGT, participants choose between four decks of cards with different frequencies of gains and losses, and learn to select from advantageous decks. Research evidence from patients with lesions of limbic structures, neurological diseases or psychological disorders has emphasized the importance of emotional processes in deciding advantageously during IGT performance (e.g. Bechara, 2004; Bechara, Damasio, Damasio, & Lee, 1999; Fellows & Farah, 2005; Goudriaan, Oosterlaan, de Beurs, & van den Brink, 2005). It is postulated that somatic markers develop through implicit learning, which marks the decks with a negative or positive valence, depending on the outcome of previous choices. These somatic markers inform explicit knowledge and facilitate learning of deck contingencies (Bechara, 2004). Thus, emotions can be beneficial to decision making when they are integral to the task. Importantly, studies of clinical populations with damage to the prefrontal cortex revealed that the absence of anticipatory makers are associated with poor performance on IGT (Bechara et al., 1994; Bechara et al., 1999).

Consistent with the SMH proposals, evidence from clinical studies focused on lesions to the VMpfc and bilateral damage to amygdala shows that an absence of physiological activity and the failure to develop somatic markers impairs decision making (e.g. Bechara et al., 1994; Bechara, Tranel, Damasio, & Damasio, 1996; Bechara, et al., 1999; Bechara et al., 2000; Tranel, Bechara, Damasio, & Damasio, 1996). The original papers reported that absence of anticipatory skin conductance responses (aSCRs) are associated with poor IGT performance in patients with lesions to the VMpfc (Bechara et al., 1994, 1996). The patients with VMpfc damage did not generate aSCRs prior to selecting from disadvantageous decks, while healthy controls did (Bechara et al., 1994, 1996, 1997). This provides support for the SMH framework that the absence of aSCRS (somatic markers) leads to poor learning, and consequently poor performance on the IGT. Consistent with these results, patients with damage to the amygdala exhibit a similar behaviour pattern and inability to develop somatic markers for disadvantageous options on the IGT (Bechara et al., 1999). Thus, clinical studies have shown patients to have no or very little aSCR activity, while healthy controls demonstrate aSCR activity that is associated with good IGT performance. The SMH is thus predicated on healthy participants exhibiting aSCRs whenever a somatic marker is expected be present – a failure to demonstrate this effect on a task devised to test the SMH would be a fundamental challenge to the theory.

Studies that measure psychophysiological data have, to some extent, provided evidence of elevated aSCRs to disadvantageous decks (e.g. Bechara et al., 1999; Bechara & Damasio, 2002). The results also indicated that aSCR may be reliable in predicting IGT performance. However, Suzuki, Hirota, Takasawa, and Shigemasu (2003) argued that the SCR to feedback (post-selection) is more influential on IGT performance than aSCR. Their results suggest that the SCR to feedback is more important to IGT performance than SCR but less so when contingencies have been learned. They found no relationship between aSCRs and overall performance. In contrast to Suzuki et al. (2003), Carter and Smith-Pasqualini (2004) related strong anticipatory somatic markers to optimal decision making and faster learning. No correlation between the SCR to feedback and optimal performance was found. The results of these studies indicate that aSCRs are associated with optimal performance on IGT; however, the direction of this association is unclear. Furthermore, Tomb, Hauser, Deldin, and Caramazza (2002) demonstrated the importance of elevated aSCRs to advantageous deck selection, and, thus, both positive and negative feedback contribute to subsequent performance. These data show that somatic markers may serve to record long-term negative and positive consequences of a certain choice option.

Crone, Somsen, Van Beek, and Van Der Molen (2004) made a similar suggestion in a study that investigated the pattern of aSCR and heart-rate variability (HRV) on an analogue of the IGT. In three groups of participants, split between bad, good and moderate performance, they found the effect of slow HRV and high aSCRs on disadvantageous deck selection compared to advantageous decks selection for the good performance group. This was not found in the bad and moderate groups. Furthermore, larger HRV and aSCRs were observed in post-feedback (after the card is chosen) related to frequent punishment from disadvantageous decks. This indicates that deck selection rests on a positively or negatively valanced somatic marker where a bad option reflects a negative state that signals avoidance. Thus, careful examination of the timing and range of psychophysiological measurements of somatic markers is warranted.

The SMH continues to provoke debate among cognitive scientists, with more than 800 papers considering this theory and associated tasks published in 2017 (see Chiu, Huang, Duann, & Lin, 2018, for review). Thus, the IGT continues to be an important task for the study of emotional decision making. According to the SMH, the search for somatic markers involves identification of bodily responses that temporally precede cognitive representations of logically organized ideas. Since the basic premises of the SMH rest on the aSCR evidence from a clinical population it is necessary to continue investigation with regards to some of the unresolved issues related to IGT performance of a healthy population. A specific point in question is whether somatic markers inform higher order processing and whether aSCRs inform IGT performance by providing somatic markers in healthy participants. For example, early research revealed that somatic signals are generated through an implicit involuntary process, which cannot be verbalized, and precede explicit awareness (Bechara, Damasio, Tranel, & Damasio 2005). Further evidence, however, indicated that IGT performance in healthy participants is determined by an interplay between ‘high-level reasoning’ (e.g. cost-benefit analysis) and somatic markers (Schiebener, Zamarian, Delazer, & Brand, 2011), whereby analytic cognition plays a more salient role than traditionally acknowledged (e.g. Simonovic, Stupple, Gale, & Sheffield, 2017a, 2017b). While there is broad consensus that an aSCR effect is evident on the IGT, it is the interpretation and timing of these responses that is debatable.

Issues with the timing and the interpretation of psychophysiological data are highlighted by Dunn, Dagleish, and Lawrence’s (2006) review. They argued that although the SMH is an elegant account of how emotion influences decision making, it lacks sufficient corroborating evidence. There is evidence that anticipatory markers correlate with successful performance on the task (e.g. Carter & Smith-Pasqualini, 2004), and that aSCR is elevated for disadvantageous decks compared to advantageous decks (e.g. Bechara & Damasio, 2002; Crone et al., 2004). However, replication of key aSCR findings on the IGT in healthy participants is lacking. Hence, despite the wealth of literature utilising the IGT as a clinical tool, the interpretation of psychophysiological results and IGT data is complex and not without criticism. The current review systematically examines two hypotheses related to psychophysiological evidence of aSCR and IGT performance in healthy individuals using Preferred Reporting Items for Systematic Reviews and Meta-Analysis (PRISMA; Moher et al., 2009). Firstly, that the strength of aSCR correlates with successful IGT performance (Hypothesis 1); and secondly, that there are differences in aSCR for disadvantageous and advantageous decks such that aSCR is elevated for disadvantageous decks compared to advantageous decks (Hypothesis 2). Finally, the review aims to assess the quality of articles measuring aSCR during the IGT.

## Methods

### Search protocol and inclusion/exclusion criteria

Extensive searches of the following psychology databases were conducted to pinpoint research studies for inclusion: *PsycARTICLES, PsycINFO, Business Source Premier, CINAHL Plus, MEDLINE and Web of Science.* The content lists of the following key journals were also reviewed: *Cognition and Emotion; Cognitive, Affective & Behavioral Science; Psychophysiology; International Journal of Psychophysiology; Journal of Psychophysiology* and *Frontiers in Psychology*. Additionally, reference lists of included studies were examined for additional studies. Key authors were contacted to obtain details of relevant unpublished studies to reduce publication bias. Key terms (‘Somatic Marker Hypothesis’, ‘SMH’, ‘Emotional Based Learning’, ‘EBL’) were combined with terms related to psychophysiological measurements (‘Psychophysiological Response’, ‘Skin Conductance Response’, ‘SCR’, ‘Skin Resistance’, ‘SR’, ‘Autonomic Response’, ‘Heart Rate’, ‘HR’, ‘Heart Rate Variability’, ‘Galvanic Skin Response’, ‘GSR’, ‘Electrodermal Activity’, ‘EDA’) and the behavioural task (‘Iowa Gambling Task’, ‘IGT’) with a standardized protocol using Boolean rules to identify relevant literature. Searches were limited to healthy human participants.

**Fig. 1:**
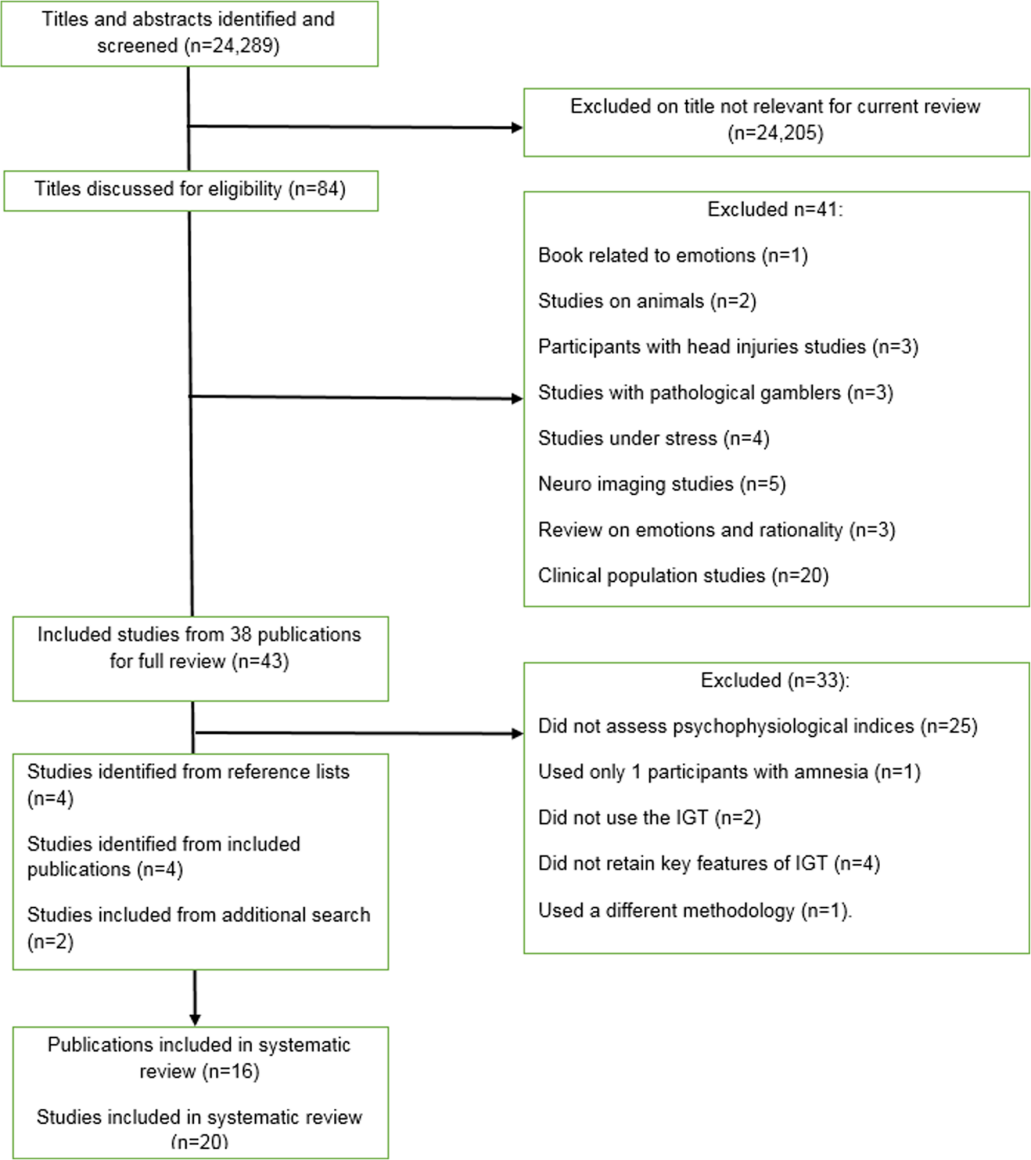
PRISMA diagram. Overview of search process, identification of studies and data extraction

English language studies that included psychophysiological measurements with IGT performance where included. Papers were excluded if they used clinical participants, if they did not use psychophysiological measurements or if they modified IGT to such an extent where the important properties of the IGT were not maintained (e.g. frequencies of reward and punishments on four decks). Electronic database searches yielded 3,999 (IGT), 244 (SMH) and 20,046 (psychophysiology measurements) results. These results were then combined generating 84 study titles that were then filtered through the search process summarized in Fig. 1. Forty-three studies were initially included for review. Thirty-three were excluded because they: had not included psychophysiological measurements (N=25); had used a clinical sample (N=1); had not used the IGT (N=2); had not retained key features of the IGT (N=4); or had used a different methodology (N=1). Four studies were identified from the reference lists of included studies. Our extensive search of aforementioned databases was undertaken in June 2015 followed by additional search in January 2019 where we identified two additional studies that were added to the corpus of studies.^1^ In total, 20 studies were included in the review.

### Quality assessment

Quality criteria were developed to assess the quality of all included studies to account for potential biases that could result from combining studies of different methodologies used, which might lead to a misleading conclusion. Quality criteria were developed based on recommendations made by the Cochrane Collaboration (2011), and included: psychophysiological measurements used, clarity of measurements taken, psychophysiological methodology and procedure used, aSCR measurements taken, feedback SCR measurements taken, IGT methodology followed, description of the study that allows replication, clear aims, appropriate analysis used, prospective power analysis, effect sizes used, information related to participants’ demographics, inclusion/exclusion criteria included, outcomes provided, and details of timing of measures. A score of 0–2 was awarded for each element (0= no details, 1= insufficient details, 2= complete details) and these were summed to give a total (0–18) (Cochrane Collaboration, 2011).

### Data extraction and synthesis

Data were extracted using a standardized extraction sheet by BS. The extraction sheet included: *identifying information* (e.g. type of study, source references and research questions); *inclusion criteria* (e.g. healthy, normal participants performing IGT while physiological measurements were taken) and *exclusion criteria* (e.g. patient population, evaluation of behavioural tests of awareness, absence of physiological measurements and different gambling tasks); *study details* (e.g. type of IGT and IGT procedure, number of participants and participants’ details such as age and ethnicity); *physiological measurements* (e.g. types and details of measurements); *results* (e.g. statistical techniques used, p values and effect sizes); *comments on the paper* (e.g. the authors comments, reviewers comments and suitability for inclusion). A second (DS) and a third reviewer (ES) independently reviewed extracted data to ensure accuracy and reliability, with reviewers meeting to confirm agreement of extraction and to establish reliability. Where there were discrepancies, these were resolved by discussion. Twenty published research studies were included in the review and evaluated for the purposes of this review. The reported studies had similar aims and utilized similar psychophysiological measurements. Nineteen studies measured skin conductance response activity (SCR), and one study measured both SCR and heart-rate variability (HRV). No study specifically measured only heart rate (HR) and HRV in relation to IGT performance.

## Results

### Participants

The 20 included studies recruited 1,364 healthy, normal participants in total. The age of participants ranged from 17 years (Carter & Smith-Pasqualini, 2004) to 85 years old (Denburg, Recknor, Bechara, & Tranel, 2006). Four studies did not report the age of participants (Guillaume et al., 2009; Visagan, Xiang, & Lamar 2012; Wagar & Dixon, 2006, 2006a). Five studies were conducted in the UK (Carter & Smith-Pasqualini, 2004; Fernie & Tunney, 2013; Jenkinson, Baker, Edelstyn, & Ellis, 2008; Visagan et al., 2012; Wright, Rakow, & Russo, 2017), six were conducted in the USA (Denburg et al., 2006; Hinson, Whitney, Holben, & Wirick, 2006, 2006a, 2006b; Hinson, Jameson, & Whitney, 2002, 2002a), two were conducted in Canada (Wagar & Dixon, 2006, 2006a) and one each was conducted in Germany (Werner, Duschek, & Schandry, 2009), Japan (Suzuki et al., 2003), Belgium (Mardaga & Hansenne, 2012), Taiwan (Yen, Chou, Chung, & Chen, 2012), France (Guillaume et al., 2009), Romania (Miu, Crisan, Chis, Ungureanu, Druga, & Vulturar, 2012) and Italy (Ottaviani & Vandone, 2014). The prevalence of female participants ranged from 30% (Suzuki et al., 2003) to 100% (Carter & Smith-Pasqualini, 2004); three studies did not report gender (Wagar & Dixon, 2006, 2006a; Yen et al., 2012). Only six studies included inclusion/exclusion criteria for study participation (Carter & Smith-Pasqualini, 2004; Denburg et al., 2006; Guillaume et al., 2009; Jenkinson et al., 2008; Werner et al., 2009; Visagan et al., 2012).

### Quality assessment of included studies

The study quality criteria were based on the total quality score derived from the quality criteria developed from the Cochrane Collaboration (2011) review. The overall quality of the studies was good (Table 1). There were procedural and methodological differences between studies and no study provided information about statistical power to detect effects. Twelve studies did not report demographic information about their participants (Guillaume et al., 2009; Hinson et al., 2006, 2006a, 2006b; Hinson et al., 2002, 2002a; Miu et al., 2012; Suzuki et al., 2003; Visagan et al., 2012; Wagar & Dixon, 2006, 2006a; Yen et al., 2012). Seven studies did not measure feedback SCR (Hinson et al., 2006, 2006a, 2006b; Hinson et al., 2002, 2002a; Denburg et al., 2006; Yen et al., 2012).

**Table 1:** Quality assessment of included studies

Quality criteria	Scores																			
Study references	1	2	3	4	5	6	7	8	9	10	11	12	13	14	15	16	17	18	19	20
Participant’s details	2	2	2	1	1	1	1	1	1	2	2	1	2	1	2	1	1	1	1	2
Inclusion/exclusion criteria	2	2	1	2	1	1	1	1	1	1	1	1	2	1	2	1	1	2	1	1
IGT procedure	2	2	2	2	1	1	1	1	1	1	1	1	2	1	2	2	2	2	1	2
Psychophysiology procedure	2	2	2	2	2	2	1	1	1	2	2	2	2	1	2	2	2	2	1	2
Exact statistic reported	2	2	2	2	2	2	2	2	2	2	2	2	2	2	2	2	2	2	2	2
Effect size	0	0	0	0	0	0	0	0	0	0	2	2	1	0	0	0	0	0	0	1
Power analysis	0	0	0	0	0	0	0	0	0	0	0	0	0	0	0	0	0	0	0	0
Reliable measure of outcome	2	2	2	2	2	2	2	2	2	2	2	2	2	2	2	2	2	2	2	2
Details of timing of measures	2	2	2	2	2	2	2	2	2	2	2	2	2	2	2	2	2	2	2	2
Total score/20	14	14	13	13	11	11	10	10	10	12	14	13	16	10	14	12	12	13	10	14

0= no details, 1= insufficient details, 2= complete details

Four studies reported using a standardised protocol for measuring SCR (Denburg et al., 2006; Fernie & Tunney, 2013; Visagan et al., 2012; Werner et al., 2009). Nine studies stated they ensured IGT protocol and procedures were followed (Carter & Smith-Pasqualini, 2004; Denburg et al., 2006; Fernie & Tunney, 2013; Guillaume et al., 2009; Jenkinson et al., 2008; Visagan et al., 2012; Wagar & Dixon, 2006, 2006a; Werner et al., 2009). Ten studies either used a modified version of the IGT or did not provide sufficient details of the IGT protocol (Hinson et al., 2002, 2002a; Hinson et al., 2006, 2006a, 2006b; Mardaga & Hansenne, 2012; Miu et al., 2012; Suzuki et al., 2003; Wright et al., 2017; Yen et al., 2012). Only two studies reported effect sizes (Mardaga & Hansenne, 2012; Miu et al., 2012)^2^.

### Method and findings

The studies used similar statistical methods to assess the research questions (e.g. ANOVA, ANCOVA and t-test), which were ascertained to be suitable for the study designed. The results are summarised in Table 2. One study used the Wilcoxon signed-ranks test (Denburg et al., 2006). Several studies’ results were associated with higher aSCRs with picks from disadvantageous decks (Mardaga & Hansenne, 2012; Guillaume et al., 2009; Wagar & Dixon, 2006; Yen et al., 2012). One of the studies reported a borderline association (Jenkinson et al., 2008), one reported no association (Denburg et al., 2006) and six studies did not separately report aSCRs for disadvantageous and advantageous decks. Significant interactions were found between aSCR amplitude and IGT performance in six studies (Carter & Smith-Pasqualini, 2004; Guillaume et al., 2009; Mardaga & Hansenne, 2012; Miu et al., 2012; Wagar & Dixon, 2006, 2006a), while one study found no interaction (Fernie & Tunney, 2013). One study associated aSCRs with picks from advantageous decks but not disadvantageous decks (Denburg et al., 2006). High SCR responses were evident in one study after encountering feedback from a punishment/reward sequence (Suzuki et al., 2003). Results from two studies suggested an interdependency between conscious knowledge and the appearance of somatic markers (Guillaume et al., 2009; Hinson et al., 2002). However, Hinson et al. argued that conscious knowledge suppresses the development of somatic markers, while in Guillaume et al.’s study performance correlated with both aSCRs and conscious knowledge. One study found that aSCR is not necessary to succeed on IGT: in the absence of a significant SCR, participants still learnt and selected advantageously (Fernie & Tunney, 2013). Hinson et al. (2006) showed that pre-experimental emotion-laden words briefly held in working memory influence deck choices: Participant choices were facilitated by the pre-existing affective reaction, whereby a positive affective load enhanced quality of decision making, and negative load reduced the quality. Finally, one study found no direct relationship between the SCR and IGT performance (Visagan et al., 2012). Their *post hoc* analysis, however, revealed that the SCR parameters were significantly related to threat anxiety and emotion regulation, which were in turn associated with IGT performance.

**Table 2:** Characteristics of included studies (N=20)

Study ID and reference	Participants’ demographics	Type of IGT	Psychophysiological measurements	Outcome
(1) Carter & Smith-Pasqualini (2004) UK	30 healthy women, aged 17–53 y (mean = 29.7, SD = 8.39)	Bechara et al. (1994). Two conditions: fake vs. real money	Performance and anticipatory SCR interaction (IGT scores as DV); SCR and learning rate per block	Correlation between aSCR and money won on IGT **
(2) Denburg et al. (2006) USA	80 healthy, older adults, aged 56– 85 y. 40 participants sampled from the previous study	Bechara et al. (2000)*^3^	SCR anticipative advantageousness in two different groups	Effect of aSCR for disadvantageous decks in one group ***
(3) Fernie & Tunney (2013) UK	32 post-graduate students, 16 males (mean age 25.68 y, SD = 1.22)	Bechara et al. (1994)*. Knowledge probed (Maia & McClelland 2004)	SCR anticipative response amplitude (effect of decks); SCR-awareness interaction	No effect of aSCR on IGT. Effect of knowledge of the task contingencies **^4,^ ***^5^
(4) Guillaume et al. (2009) France	30 participants (11 male)	Bechara et al. (1994)*. Knowledge probed (Maia & McClelland 2004)	Anticipatory SCR; Performance and beneficial SCR interaction (IGT scores as DV)	Effect of aSCR for disadvantageous deck selection **^,^ ***
(5) Hinson et al. (2002) USA	Study 2: 45 students, aged 18–24 y. 58% female	Bechara et al. (1994)*. Overall payoffs are less extreme	Anticipatory SCR; SCR amplitude as DV	aSCRs predicted IGT performance **
(6) Hinson et al. (2002a) USA	Study 3: 47 students, aged 18–24 y. 58% female	Bechara et al. (1994)*. Overall payoffs are less extreme	Anticipatory SCR; SCR amplitude as DV	No effect of aSCR. Working memory connected to the development of somatic markers **
(7) Hinson et al. (2006) USA	Study 1: 70 students, aged 18–25 y. 60% female	Bechara et al. (1994)*. Affective reaction manipulated	SCR and learning rate per block. Positive and negative (emotionally charged) word load was used and related to learning rate per block	Effect of aSCRs for advantageous decks. SCR amplitude appeared after IGT performance was already well established **
(8) Hinson et al. (2006a) USA	Study 2: 40 students, aged 18–25 y. 55% female	Bechara et al. (1994)*. Affective reaction manipulated	SCR and learning rate per block. Positive and negative (emotionally charged) word load was used and related to learning rate per block	Effect of aSCRs for advantageous decks **
(9) Hinson et al. (2006b) USA	Study 3: 70 students, aged 18–25 y. 60% female	Bechara et al. (1994)*. Affective reaction manipulated	SCR and learning rate per block. Positive and negative (emotionally charged) word load was used and related to learning rate per block	Effect of feedback SCR (post deck selection). SCR effect on IGT score but not a major factor **
(10) Jenkinson et al. (2008) UK	41 healthy individuals aged 18–28 y (M = 20.5, SD = 2.8; 11 male, 30 female)	Bechara et al. (1994)*. Real and fake money versions	Anticipatory and appraisal SCR; Performance and beneficial SCR interaction (IGT scores as DV)	The borderline significant effect for aSCR rises preceding disadvantageous decks selections ***
(11) Mardaga & Hansenne (2012) Belgium	32 healthy participants (10 men) aged 19–34 y (mean = 22.9, SD = 4.03) students	Bechara et al. (1994)*. Gains and losses presented sequentially, as opposed to the original parallel presentation	Anticipatory and Appraisal SCR; SCR amplitude; Performance and beneficial SCR interaction (IGT scores as DV)	Effect of aSCR for disadvantageous decks selection **
(12) Miu et al. (2012) Romania	135 students, (118 women) aged 16-42 y, (M= 21.6)	Bechara et al. (1994)*	SCR anticipative response amplitude; Performance and beneficial SCR interaction (IGT scores as DV)	Effect of aSCR. SCR mediated IGT performance **
(13) Ottaaviani & Vandone (2015) Italy	445 healthy participants (348 men, 97 women) employed in management societies	Bechara et al. (1994)*	Anticipatory and Appraisal SCR; SCR amplitude for disadvantageous decks^6^	No effect of aSCR on IGT **. Effect of aSCR for disadvantageous decks ***
(14) Suzuki et al. (2003) Japan	40 students, (27 men and 13 women) aged 18–23 y, (M= 19.9, SD= 1.29)	Bechara et al. (1994)*	Anticipatory and appraisal SCR	No effect of aSCR. Effect of feedback SCR (post deck selection) **^,^ ***
(15) Visagan et al. (2012) UK	33 students, (15 men, 18 women) aged 20–40 y, (M= 22.2, SD= 3.7)	Bechara et al. (1997)*	Anticipatory SCR; SCR and learning rate per block	No effect of aSCR ***
(16) Wagar & Dixon (2006) Canada	12 undergraduate students	Bechara et al. (1994)*	SCR anticipative disadvantageousness; Performance and anticipatory SCR interaction (IGT scores as DV)	Effect of aSCR for disadvantageous decks **^,^ ***
(17) Wagar & Dixon (2006a) Canada	12 undergraduate students.	Bechara et al. (1994)*	SCR anticipative disadvantageousness; Performance and beneficial SCR interaction (IGT scores as DV)	Effect of aSCR for disadvantageous decks **^,^ ***
(18) Werner et al. (2009) Germany	64 students, (32 men, 32 women)	Bechara et al. (1994)*	SCR and HR anticipative response amplitude; SCR and HR appraisal response amplitude	No correlation between aSCR and IGT **
(19) Yen et al. (2012) Taiwan	34 undergraduate students (11 males, 23 females), aged 20–31 y	Bechara et al. (1999)*	SCR anticipative disadvantageousness (interaction with expected risk; intuition and conceptual phases)	Effect of aSCR and deck choices
(20) Wright et al. (2017) UK	72 undergraduate students (18 males, 54 females), aged 18–21 y	Bechara et al. (1999)*	Anticipatory and appraisal SCR; SCR amplitude for advantageous and disadvantageous decks	Effect of aSCR and deck choices ** No effect of aSCR for disadvantageous decks ***

^3^Note all * are the computerised version

^4^Note all ** Testing hypothesis 1 (relationship between overall aSCR and IGT performance)

^5^Note all *** Testing hypothesis 2 (differences between good and bad decks

^6^The authors recorded anticipatory SCRs before the disadvantageous decks during the first 40 and the last 60 trials. They provided the data that were calculated to suit our analyses and our hypotheses.

There were differences in timing and quantification of SCR peak amplitudes: 11 studies defined anticipatory responses within a 5-s window before deck selection (Fernie & Tunney, 2013; Guillaume et al., 2009; Hinson et al., 2006; Hinson et al., 2006a; Hinson et al., 2006b; Jenkinson et al., 2008; Miu et al., 2012; Visagan et al., 2012; Wagar & Dixon, 2006; Wagar & Dixon, 2006a; Yen et al., 2012). Three studies used 5-s aSCR and 10-s feedback SCR for defining responses (Hinson et al., 2002; Hinson et al., 2002a; Mardaga & Hansenne, 2012). Three studies defined SCR responses within 1–7 s (Werner et al., 2009), 1–9 s (Carter & Smith-Pasqualini, 2004) and 10 s (Suzuki et al., 2003) before and after the participants chose a deck. Fourteen studies quantified SCR peak as a mean response within their proposed time frame and then averaged mean amplitudes across 100 trials (Fernie & Tunney, 2013; Guillaume et al., 2009; Hinson et al., 2002; Hinson et al., 2002a; Hinson et al., 2006; Hinson et al., 2006a; Hinson et al., 2006b; Jenkinson et al., 2008; Mardaga & Hansenne, 2012; Miu et al., 2012; Yen et al., 2012; Wagar & Dixon, 2006; Wagar & Dixon, 2006a; Werner et al., 2009). In contrast, three studies calculated the largest SCR amplitude or the first SCR peak amplitude and designated the responses as aSCR or feedback SCR (Carter & Smith-Pasqualini, 2004; Suzuki et al., 2003; Visagan et al., 2012).

Fourteen out of the 15 studies that are included in first meta-analysis calculated a beneficial autonomic response by subtracting aSCR scores for advantageous decks (C+D) from aSCR scores for disadvantageous decks (A+B) that was correlated with IGT performance ([1] Carter & Smith-Pasqualini 2004; [4] Guillaume et al., 2009; [5] Hinson et al., 2002; [6] Hinson et al.,2002a; [7] Hinson et al., 2006; [8] Hinson et al., 2006a; [9] Hinson et al., 2006b; [11] Mardaga & Hansenne, 2012; [12] Miu et al., 2012; [13] Ottaaviani & Vandone, 2015; [14] Suzuki et al., 2003; [16] Wagar & Dixon, 2006; [17] Wagar & Dixon, 2006a; [18] Werner et al., 2009) and one study calculated aSCR responses separately for advantageous and disadvantageous decks ([20] Wright et al., 2017). Eight studies, which are included in second meta-analysis, examined differences in aSCR scores before the advantageous picks (C+ D) and the disadvantageous picks (A + B) ([3] Fernie & Tunney, 2013; [4] Guillaume et al., 2009; [10] Jenkinson et al., 2009; [13] Ottaaviani & Vandone, 2015; [14] Suzuki et al., 2003; [16] Wagar & Dixon, 2006; [17] Wagar & Dixon, 2006a; [20] Wright et al., 2017).

### Meta-analyses

All analyses were performed using Meta-Essentials (Suurmond, van Rhee, & Hak, 2017). The meta-analysis calculator was used to compute R statistics (Lyons, 2004). Three studies were excluded, one based on the consistency of IGT and SCR methods (Yen et al., 2012) and two because of insufficient data (Denburg et al., 2006; Visagan et al., 20125). Seventeen studies were included in the meta-analysis. Two separate meta-analyses were performed testing Hypothesis 1 (the relationship between overall aSCR and IGT performance; k=15) and Hypothesis 2 (greater aSCR for bad decks compared to good decks; k=8). In addition, the total risk of bias score was used as a potential moderator in the meta-analysis.

### Anticipatory SCR and IGT performance

**Fig. 2:**
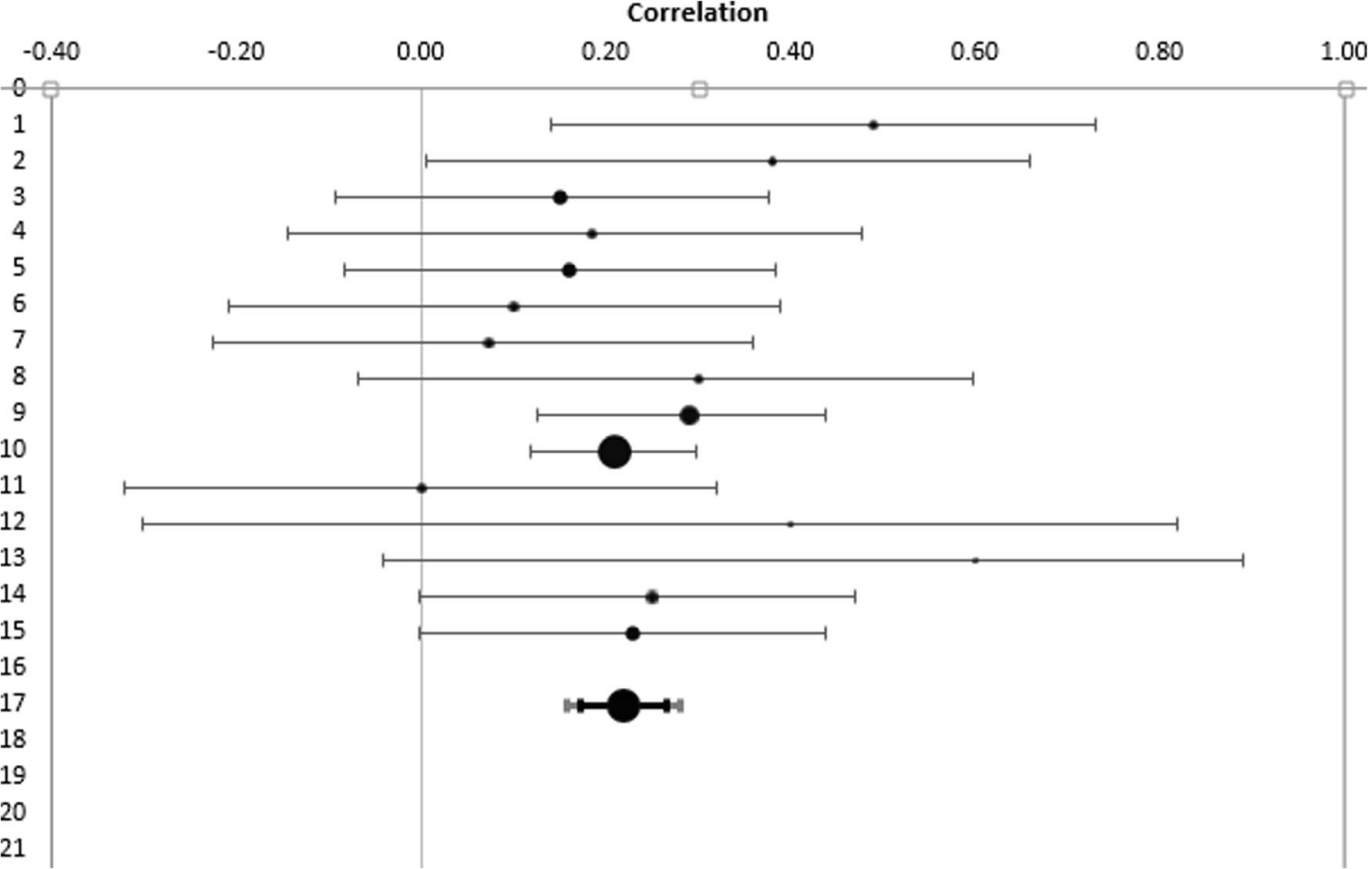
Forest plot. Combined effect size confidence intervals of studies correlating aSCR and successful IGT performance

**Fig. 3:**
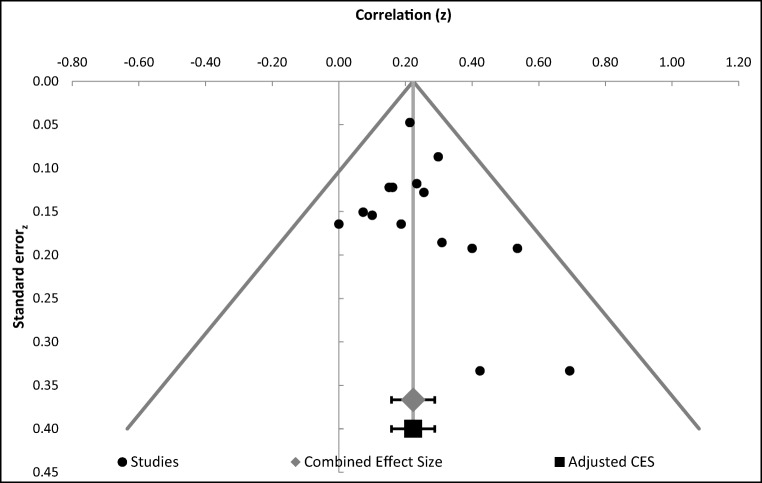
Funnel plot of standard error by effect size for studies correlating aSCR and successful IGT performance

First, analyses were conducted and effect sizes calculated for each study (Table 3). Specifically, we calculated r and confidence interval (CI; upper and lower) for studies that tested an effect of aSCRs in relation to IGT performance. Then a combined effect size was calculated and examined by using a Forest plot (Fig. 2). The Forest plot revealed a combined effect size of r = 0.22 (CI 0.17–0.27, p<0.00001) representing a small to medium relationship between aSCR and IGT performance (Table 4) such that SCR before deck picks correlated positively with the proportion of good deck selections. The overall effect size was also homogenous, Q (15) = 11.04, p<.0001; I^2^ = 0.00, indicating that there was no heterogeneity issue. Publication bias analyses were undertaken by calculating fail-safe N (Rosenthal, 1979). Moreover, study quality was not a significant moderator (z = 0.93, *p*=0.35). The fail-safe N was 247, suggesting that even if a greater number of additional relevant studies with null results were included, the overall effect size would remain significant. However, because fail-safe N is biased towards overestimating the number of null studies required to render the overall effect size nonsignificant (Carson, Schriesheim, & Kinicki, 1990), a funnel plot of the standard error by the standard mean differences was generated (Fig. 3). The distribution was symmetrical, further suggesting no issues regarding publication bias.

**Table 3:** Effect sizes of studies included in the meta-analysis related to aSCR correlates with successful IGT performance

Study	N	R	95% CI	Weight
Carter & Smith-Pasqualini (2004)	30	.49*	.14, .73	2.45%
Guillaume et al. (2009)	30	.38*	.01, .66	2.45%
Hinson et al. (2002)†	45	.10*	-.21, .39	3.81%
Hinson et al. (2002a) †	47	.07*	-.23, .36	3.99%
Hinson et al. (2006) †	70	.15*	-.09, .38	6.08%
Hinson et al. (2006a) †	40	.19*	-.14, .48	3.36%
Hinson et al. (2006b) †	70	.16*	-.08, .38	6.08%
Mardaga & Hansenne (2012)	32	.30*	-.07, .60	2.63%
Miu et al. (2012)	135	.29*	.13, .44	11.98%
Ottaaviani & Vandone (2015) †	445	.21	.12, .30	40.11%
Suzuki et al. (2003)	40	.00	-.32, .32	3.36%
Wagar & Dixon (2006)	12	.40*	-.30, .82	0.82%
Wagar & Dixon (2006a)	12	.60*	-.04, .89	0.82%
Werner, Duschec & Schandry (2009)	64	.25	.00, .47	5.54%
Wright et al. (2017)	72	.23*	.00, .44	6.53%

*All p values significant at .05

†All effect sizes provided by the author

**Table 4:** Summary of meta-analysis related to aSCR correlates with successful IGT performance

All studies	K	N	Combined effect 95%		Combined z	Combined	I^2^
			size (r)	CI		p	
Homogeneous	15	1,147	.22	.17 to .27	7.40	p< .0001	.00

### Differences in anticipatory SCR between disadvantageous and advantageous decks

**Fig. 4:**
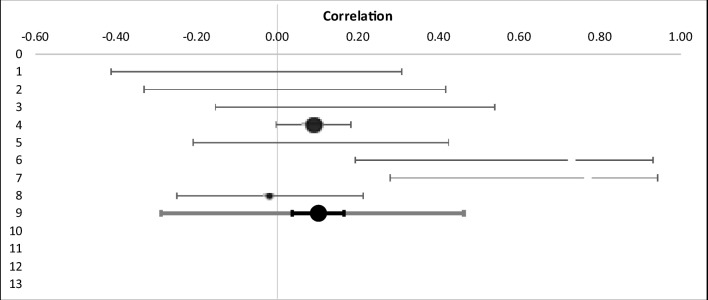
Forest plot. Combined effect size confidence interval related to aSCR differences between the disadvantageous and advantageous decks

**Fig. 5:**
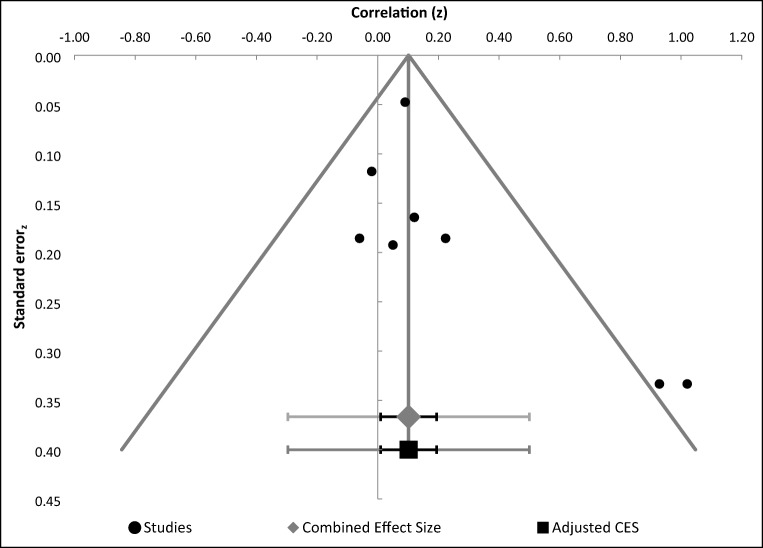
Funnel plot of standard error by effect size for studies correlating aSCR differences between the disadvantageous and advantageous decks

First, effect sizes were calculated for each study (Table 5). Specifically, we calculated r and CI (upper and lower) for studies that found an effect of anticipatory SCRs in relation to disadvantageous deck picks. Then a combined effect size was calculated and examined using a Forest plot (Fig. 4). The aSCR before the disadvantageous deck picks were higher than before the advantageous deck picks. The Forest plot revealed a combined effect size of r = 0.10 (CI 0.04–0.16, p=0.005) representing a small effect (Table 6). The overall effect size was not homogenous, Q (8) = 16.15, p=.005; I^2^ = 0.03, indicating low homogeneity. Indeed, inspection revealed two studies, both authored by Wagar and Dixon (2006, 2006a), found medium to large difference, whereas the other six studies found either small differences (Guillaume et al., 2009; Ottaaviani & Vandone 2015) or no differences (Fernie & Tunney, 2013; Jenkinson et al., 2009; Suzuki et al., 2003; Wright et al., 2017). However, study quality was not a significant moderator (z = -0.92, *p*=0.36). Publication bias analyses were undertaken first by calculating fail-safe N (Rosenthal, 1979). The fail-safe N was 26, suggesting that if a relatively small number of additional relevant studies with null results were included, the overall effect size would not remain significant. However, because fail-safe N is biased towards overestimating the number of null studies required to render the overall effect size nonsignificant (Carson et al., 1990), a funnel plot of the standard error by the standard mean differences was generated (Fig. 5). The distribution is not symmetrical, confirming issues regarding publication bias.

**Table 5:** Effect sizes of included studies related to aSCR differences between the disadvantageous and advantageous decks

Study	N	R	95% CI	Weight	
Fernie & Tunney (2013)	32	-.06	-.41, .31	4.43%	
Guillaume et al. (2009)	30	.05*	-.36, .36	4.13%	
Jenkinson et al. (2009)	32	.22	-.15, .54	4.43%	
Ottaaviani & Vandone (2015) †	445	.09*	.00, .18	67.58%	
Suzuki et al. (2003)	40	.12	-.15, .54	5.66%	
Wagar & Dixon (2006)	12	.73*	.19, .93	1.38%	
Wagar & Dixon (2006a)	12	.77*	.28, .94	1.38%	
Wright et al. (2017)	72	.02	-.25, .21	11.01%	

*All p values significant at .05

†All effect sizes provided by the author

**Table 6:** Summary of meta-analysis related to aSCR differences between the disadvantageous and advantageous decks

All studies	K	N	Combined effect 95%		Combined z	Combined	I^2^
			size (r)	CI		p	
Homogeneous	8	678	.10	.04 to .16	2.60	p= .005	.03

## Discussion

This systematic review identified 20 studies measuring aSCR to IGT in healthy populations. All of the studies included in the systematic review used aSCR measurements and predominantly Bechara et al.’s (1994) original IGT. Seventeen studies were included in two meta-analyses testing the overall aSCR effect on IGT performance differences, and aSCR responses between the good and the bad deck picks. The first meta-analysis revealed a small to medium significant relationship between aSCR and the proportion of good deck selections. The results provide support for the SMH; however, the effect size indicated that other factors are important during decision making. The second meta-analysis revealed a small, significant effect of aSCR between the bad and the good deck picks. However, the overall effect size was not homogenous, and the distribution was not symmetrical, indicating that there is no clear aSCR distinction between the bad and the good decks because of heterogeneity and possible publication bias.

### Meta-analysis 1: The relationship between overall aSCR and IGT performance

The results from the first meta-analysis indicate that overall aSCR correlates with successful performance on the IGT (Carter & Pasqualini, 2004; Guillaume et al., 2009; Mardaga & Hansenne, 2012; Miu et al., 2012; Wagar & Dixon, 2006; Werner et al., 2009). However, only four studies reported medium and large effect sizes (Carter & Pasqualini, 2004; Guillaume et al., 2009; Wagar & Dixon, 2006; Wagar & Dixon, 2006a), whereas eight out of the remaining 11 studies found small, significant differences. It is notable that the four studies reporting medium and large effect sizes had small sample sizes; however, the quality rating of the studies was good and unrelated to effect size; they reported psychophysiological and IGT procedures in good detail. Conversely, six out of the other eleven studies scored slightly lower on the quality assessment with few points lost on the clarity of physiological and IGT procedures. For example, they did not report procedures of adapted IGTs in good detail (Hinson et al., 2002, 2002a; Hinson et al., 2006, 2006a, 2006b; Suzuki et al., 2003); and/or did not report the exact physiological procedures (Hinson et al., 2006; Hinson et al., 2006a; Hinson et al., 2006b; Suzuki et al., 2003). Thus, methodological differences may account for the differing strength of the findings, whereas overall quality did not.

While there is agreement that there is a correlation between the strength of overall aSCR signals and IGT performance, it is difficult to differentiate what aSCRs for bad and good decks represent. Dunn et al.’s (2006) review pointed out that the aSCR signal may be “a response to feedback, an indicator of risk, a marker of post-decision emotion state, or a signal of how good or bad a particular response option is” (p. 251). Furthermore, the absence of the aSCRs signals can lead to a good IGT performance, and aSCR activity is not necessary to succeed on the IGT (Fernie & Tunney, 2013). Fernie and Tunney (2013) showed that the participants learn to select advantageously on the IGT and develop knowledge of the task contingencies sufficient to guide behaviour after approximately 40 trials without developing a SCR. Furthermore, the post-knowledge difference in feedback SCRs indicates that the choices could have been made based on conscious knowledge. This is in line with the suggestion that performance on IGT may be guided by two pathways: aSCRs may represent somatic markers that guide successful decision making during the IGT (Bechara et al., 1996), or aSCRs represent a proxy of good performance and are caused by conscious knowledge (e.g. Guillaume et al., 2009; Maia & McClelland, 2004; Wagar & Dixon, 2006). This indicates a complex interplay between the emotion-based signals and conscious knowledge during the task, and the relatively slow time course of aSCR signals make it difficult to separate the different influences on aSCR signal. Hence, unless participants are having differential aSCRs to good and bad choices, it is questionable if a magnitude of an absolute somatic marker guides decision making (e.g. Maia & McClelland, 2004).

### Meta-analysis 2: Differences between aSCR for good and bad decks

The results from the second meta-analysis of eight studies indicate that there is a difference in aSCRs between disadvantageous and advantageous deck selection: aSCR is elevated for disadvantageous decks compared to advantageous decks. However, that difference is small and due to heterogeneity in the findings the results may be misleading, such that: (a) some of the studies in the meta-analysis overestimated the true effect size because they are based on a biased sample or a small sample size (e.g. Egger, Davey Smith, Schneider, & Minder 1997); and/or (b) there could be a number of studies missing (published/unpublished). Indeed, two smaller studies (Wagar & Dixon, 2006, 2006a) found medium to large differences, two larger studies found small differences (Guillaume et al., 2009; Ottaaviani & Vandone, 2015), and four studies found small, non-significant differences; there are no other clear methodological differences that may account for their different findings. Indeed, the quality rating of the studies was good and was unrelated to effect size; studies reported psychophysiological and IGT procedures in good detail. It may be that aSCR measurements do not differentiate between the positive and negative aSCRs, and so these studies cannot provide definitive data about the valence of the aSCR signals. This is in accordance with the argument that SCR is influenced by the activation of the neuropsychological, behavioural, inhibitory system implicated in responses to punishment and frustration from a lack of reward. It is therefore difficult to interpret SCR as being based on negative outcomes alone (e.g. Fowles, 1988).

It should be noted that some studies reported aSCR differences in response to disadvantageous deck selection but not advantageous deck selection (e.g. Guillaume et al., 2009; Ottaaviani & Vandone, 2015; Wagar & Dixon 2006). This is consistent with Damasio’s (1994) original proposition that aSCRs for disadvantageous decks lead to a shift in preference from bad to good decks. However, there was evidence of a correlation between the aSCR related to the advantageous deck selection and successful performance on the task (e.g. Denburg et al., 2006, Hinson et al., 2006). This raises the possibility that it is not the intensity of aSCRs signals before the bad decks that is important, but the contrast between aSCR signals before good versus bad decks (e.g. Guillaume et al., 2009). However, further studies are needed to resolve this issue. Interestingly, only eight out of 20 studies examined aSCR differences between the good and the bad decks. Considering that the key argument in support of the SMH emphasises the importance of anticipatory changes related to the disadvantageous decks in learning IGT, we would encourage researchers to examine this effect in more detail.

The interpretation of our results may be complicated by a number of factors. Differences in aSCRs between decks found on the IGT may be confounded by expectancies about reward and punishments after a deck has been chosen rather than an anticipatory signal indicating deck quality (e.g. Hinson et al., 2006, 2006a; Wagar & Dixon, 2006). This raises the possibility that anticipatory somatic markers reflect the outcome of a decision process and serve to ready the subject for new information when making an uncertain response,

or, alternatively, are driven by the risk or punishment presentation associated with a specific deck (e.g. Davis, Love, & Maddox, 2009; Suzuki et al., 2003). Suzuki et al. (2003) suggested that feedback SCR, rather than aSCR of deck selection, is more important for mediating IGT performance. This feedback response was related to punishment encountered when choosing from the disadvantageous decks (Suzuki et al., 2003). This is in line with a view that anticipatory markers reflect the outcome of a decision involving a significant or uncertain choice (Davis et al., 2009). Indeed, if stronger feedback SCR follows selections from disadvantageous decks (Jenkinson et al., 2008) and frequent punishments from both decks (Mardaga & Hansenne, 2012), then there is a possibility that somatic markers arise for the outcome of deck choices (Davis et al., 2009). This is important since these results relate optimal performance on the IGT to participants’ expectation of punishments and rewards only after a deck has been selected. Furthermore, this also challenges the SMH view that anticipatory somatic markers serve as inputs to the decision process and code the value or risk associated with each deck.

In summary, the results from both meta-analyses suggest that aSCR is a somatic marker in IGT studies. Nonetheless, we cannot exclude the possibility that somatic markers can be imperfectly represented by SCR. The somatic markers may represent an anticipated, affective reaction before the choice had been made; however, they may also represent an affective reaction after the choice had been made and when a person has enough knowledge of the task to predict a choice. Thus, the somatic marker may be a correlate of good performance rather than a cause of it.

### Methodological and measurement issues

Our review indicates that there may be an issue when quantifying aSCR and feedback SCR measurements. Levinson, Edelberg, and Bridger (1984) suggest that any SCR that begins between 1 and 3 s following stimulus onset may be elicited by the stimulus. This latency effect is followed by SCR rise and recovery time. One issue when quantifying aSCRs is whether a response is elicited prior to recovery from a preceding response. Thus, the amplitude of the second response may be distorted by being superimposed on the recovery of the first response. This makes interpreting aSCR and feedback SCR difficult, and emphasizes the utility of logarithmic data transformation to remedy this issue and/or the need for sufficient latency between trials to avoid response distortion. Indeed, Lykken and Venables (1971) proposed standardised techniques for SCR measurement where the correction procedure (e.g. computing the logarithm of SCR) can significantly reduce errors in measurements. Although most studies reviewed here have logarithmically transformed the aSCR amplitude to fit normal distribution, it is noticeable that the aSCR response time, included in the logarithmic transformation, differs between the studies; for example, Mardaga and Hansenne (2012) used 5-s aSCR and 10-s feedback SCR for defining responses, whereas Werner et al. (2009) defined aSCR responses within 1–7 s, and this may have generated some measurement errors. Furthermore, it has been noticed that variations in room temperature and handwashing with soap and water may create errors in SCR measurements (Venables & Christie, 1973). Venables and Christie (1973) recommended handwashing with nonabrasive soap before having the electrodes attached and a constant room temperature of 23°C. However, only two studies reported that they had controlled room temperature (Guillaume et al., 2009; Mardaga & Hansenne, 2012) and none of the studies reported participants hand washing with nonabrasive soap. Accordingly, the meta-analysis may underestimate the relationship between aSCR and IGT performance.

The measurement choice of SCR in the studies in this meta-analysis as an indicator of somatic markers might in itself be problematic. This is because SCR might not involve regulation by the autonomic nervous system, but rather represent regulation by the brain stem and hypothalamus. A better measure for sustained tonic levels of tension may be skin-conductance level, which measures sweat gland activity in response to events, but not the SCRs, which measure transient responses (e.g. Marr, 2011). Tonic levels of muscular tension produced under continuous choice alternatives are generally known to modulate avoidance behaviours, and SCR has been found to be an effective predictor of rejecting unpleasant and psychologically distressing behavioural options (van‘t Wout, Kahn, Sanfey, and Aleman, 2006).

The quality of studies included in the review was good but is not without limitation. There were some procedural and methodological differences between studies. For example, some studies reported using a standardised protocol for measuring SCR (Denburg et al., 2006; Fernie & Tunney, 2013; Werner et al., 2009; Visagan et al., 2012), whereas most of the studies did not provide sufficient details of the protocol to allow replication. Furthermore, several studies calculated a beneficial autonomic response where the summed aSCR scores for advantageous decks (C+D) were subtracted from scores on disadvantageous decks (A+B), to calculate absolute aSCRs (Guillaume et al., 2009; Mardaga & Hansenne, 2012; Miu et al., 2012). This might create an issue when interpreting somatic markers because an absolute aSCR may be subject to many superfluous influences that have nothing to do with discrimination of choice outcomes in the IGT (e.g. Dawson, Schell, & Filion, 2000). For example, the absolute magnitude of aSCR choices may depend on the features of the payoff schedules, and not the category of good or bad choice per se (e.g. Hinson et al., 2002). Thus, it is difficult to fully assess the quality of all included studies and to account for potential biases that result from different protocol and/or outcome measures used, which might impact on the conclusions drawn.

## Future research

Further investigation of somatic markers and the associated brain structures that represent the state of the body is warranted, especially the right-hemisphere insula. A meta-analysis of neuroimaging studies of the IGT may also be prudent. Clarification is also needed about the role of aSCRs when there are differing levels of knowledge (e.g. low uncertainty associated with risk vs. high uncertainty associated with ambiguity). Heightened arousal can have an adaptive role when there is sufficient knowledge about the outcomes (de Berker et al., 2015; FeldmanHall, Glimcher, Baker, & Phelps, 2016), and therefore stronger aSCR signals should be related to the later stages of the IGT contrary to the SMH prediction. This raises the possibility that aSCRs during the initial stage of the IGT reflect some aspects of cognitive processes rather than high uncertainty associated with ambiguity. This accords with the view that arousal that accompanies choice under uncertainty develops through a ‘cognitive’ assessment of the chosen action and its consequences (Otto, Knox, Markman, & Love, 2014).

Future research should consider using measures that afford greater precision, including heart rate and blood pressure with an electrocardiogram (e.g. Crone et al., 2004) or pupil dilation. This is important because the aSCR signal during the IGT performance may be part of a broader response including attentional bias and implicit learning (e.g. Turnbull, Bowman, Shanker, & Davies, 2014) that can be measured by eye tracking. For instance, Bierman, Cleeremans, Ditzhuyzen, and van Gaal (2004) used the eye-tracking methodology to explicate the processes involved in the development of somatic markers. Furthermore, anticipatory pupil dilations may predict IGT performance (Simonovic et al., 2017b) and may be used as a marker of noradrenaline activity, which has been associated with reward prediction errors (Lavin, San Martin, & Jubal, 2014). These studies suggest that somatic marker activity could be extended to other domains of arousal that might provide a better assessment of the SMH.

## Conclusion

This review provides some evidence in support of the SMH with significant, small-to-medium correlations between the aSCR and IGT performance and a small difference in aSCR between the disadvantageous and advantageous decks. The quality of the evidence is good overall. However, there is a possibility that measures of skin conductance only provide a limited insight regarding how optimal deck selection is guided by somatic markers. For studies to be replicable, they need to report procedures and details of either original IGT or modified IGT tasks to reduce sources of heterogeneity and enable more robust conclusions. Hence, to conclusively demonstrate implicit learning through somatic markers, studies should be consistent in reporting the standardised protocols, methodologies and measures. We would encourage researchers to not only extend IGT tasks but to replicate findings with previous versions of the IGT and equivalent protocols.

## Footnotes

1 Note that our additional search identified four studies that could be included in the systematic review. The authors of two studies did not respond to our request for data clarification (Akiyama & Hasegawa, 2014; Sandor & Gurvit, 2019).

2 The studies were numbered as follow: (1) Carter & Smith-Pasqualini (2004); (2) Denburg et al. (2006); (3) Fernie & Tunney (2013); (4) Guillaume et al. (2009); (5) Hinson et al. (2002); (6) Hinson et al. (2002a); (7) Hinson et al. (2006); (8) Hinson et al. (2006a); (9) Hinson et al. (2006b); (10) Jenkinson et al. (2008); (11) Mardaga & Hansenne (2012); (12) Miu et al. (2012); (13) Ottaviani & Vandone (2015); (14) Suzuki et al. (2003); (15) Visagan et al. (2012); (16) Wagar & Dixon (2006); (17) Wagar & Dixon (2006a); (18) Werner et al. (2009); (19) Yen et al. (2012); (20) Wright, Rakow & Russo (2017).
